# Rapid osteoinduction of human adipose-derived stem cells grown on bioactive surfaces and stimulated by chemically modified media flow

**DOI:** 10.1186/s13036-025-00491-2

**Published:** 2025-03-14

**Authors:** Karolina Truchan, Barbara Zagrajczuk, Katarzyna Cholewa-Kowalska, Anna Maria Osyczka

**Affiliations:** 1 Department of Cell Biology and Imaging, Institute of Zoology and Biomedical Research, Faculty of Biology, Jagiellonian University, Gronostajowa St. 9, Krakow, 30-387 Poland; 2 Department of Glass Technology and Amorphous Coatings, Faculty of Materials Science and Ceramics, AGH University of Science and Technology, Mickiewicza Ave. 30, Krakow, 30-059 Poland

**Keywords:** Adipose-derived stem cells, Osteoinduction, BMP-2, Phenamil, ERK inhibitor, Zinc, Strontium, Bioactive glasses, PLGA, Cell-based bone therapies

## Abstract

Adipose-derived stem cells (ASCs) provide an ample, easily accessible source of multipotent cells, an alternative to bone marrow-derived stromal cells (BMSCs), capable of differentiating into osteoblasts. However, the osteogenic potential of ASCs is reportedly lower than that of BMSCs and protocols to effectively differentiate ASCs into osteoblasts are in high demand. Here, we present novel strategies for effective osteogenic differentiation of human ASCs by combining their culture on bioactive growth surfaces with their treatment with specific supplements in osteogenic medium and application of fluid shear stress. Human ASCs were cultured on PLGA-based composites containing 50 wt% sol-gel bioactive glasses (SBGs) from the SiO_2_-CaO±P_2_O_5_ system, either unmodified or modified with 5 wt% ZnO or SrO. The osteogenic medium was supplemented with recombinant human bone morphogenetic protein 2 (BMP-2), MEK1/2 kinase inhibitor (PD98059) and indirect Smurf1 inhibitor (Phenamil). Fluid shear stress was applied with a standard horizontal rocker. ASC culture on SBG-PLGA composites along with the osteogenic medium supplements enhanced the expression of both early and late osteogenic markers. Modification of SBG with either SrO or ZnO further enhanced osteogenic gene expression compared to ASCs cultured on composites containing unmodified SBGs. Notably, the application of fluid shear stress synergistically strengthened the osteogenic effects of bioactive composites and medium supplements. We also show that the presented culture strategies can drive ASCs toward osteoblastic cells in a 3-day culture period and provide mineralizing osteoblasts through a short, 7-day ASC preculture on bioactive composites. Our results also indicate that the applied osteogenic treatment leads to the phosphorylation of β-catenin and CREB or the COX-2 expression. We believe the presented strategies are feasible for rapid ASC differentiation to early osteoblasts or mineralizing osteoblastic cells for various potential cell-based bone regeneration therapies.

**Graphical Abstract:**

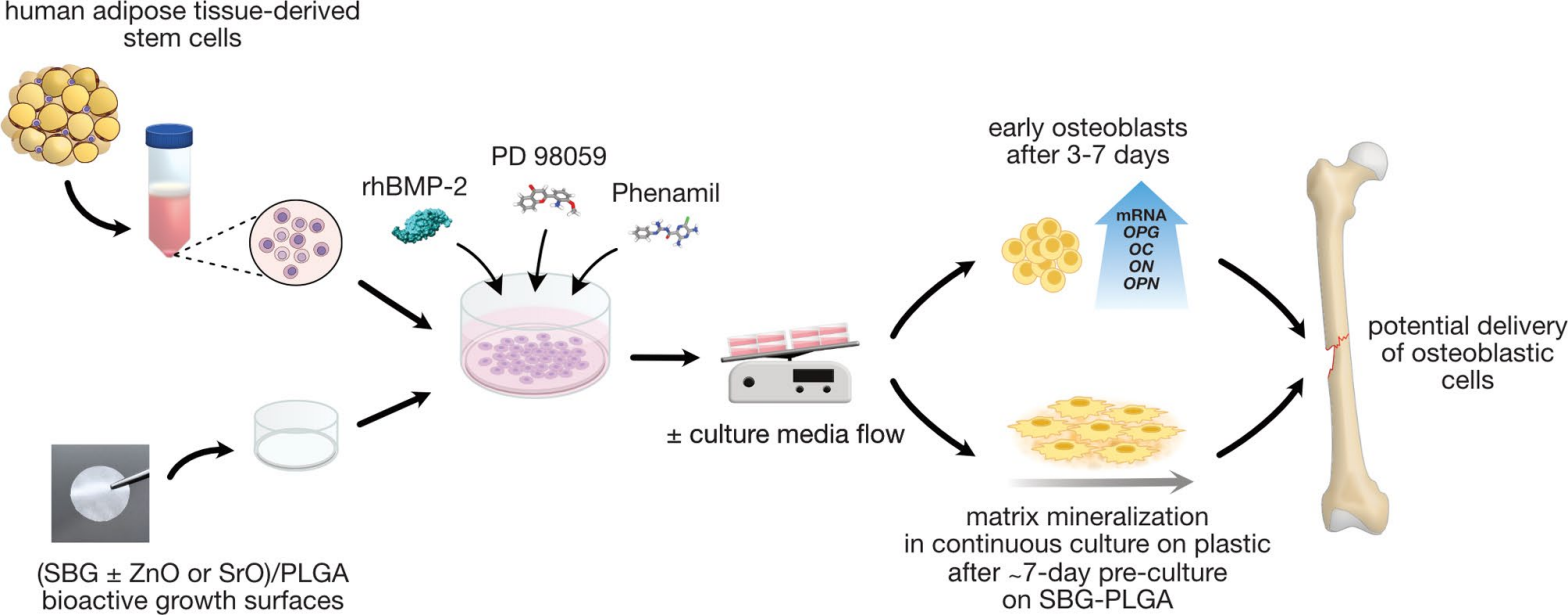

**Supplementary Information:**

The online version contains supplementary material available at 10.1186/s13036-025-00491-2.

## Background

Mesenchymal stem cells (MSCs) have also been referred to as medicinal signaling cells since Arnold Caplan modified the name in 2017 [1], while retaining the same MSC abbreviation. MSCs can be found in various adult tissues, with the most explored bone marrow-derived MSCs (BMSCs). BMSCs have been investigated for years for their contribution to bone formation processes as well as their regenerative potential in various bone-related therapies. With the discovery of MSCs in adipose tissue (ASCs) and the definition of their potential for differentiation into other phenotypes besides fat cells, such as bone-forming cells [2], these cells quickly became a viable alternative to BMSCs [3]. Human subcutaneous adipose tissue biopsies provide a greater number of adult MSCs with a minimally invasive procedure, compared with bone marrow harvests. However, it is believed that ASCs have lower bone-forming potential than BMSCs [4]. Hence, effective methods for differentiating ASCs into bone cells continue to be sought.

The delivery of functional bone cells for fractured and/or diseased bone requires at least biocompatible if not bioactive scaffolds or growth surfaces. Poly(lactic-co-glycolic) acid (PLGA), a biocompatible and biodegradable polymer that undergoes controlled biodegradation through natural pathways [5], has received approval for use in biomedical products, including bioresorbable and biodegradable sutures [6]. PLGA does not display osteoinductive properties, but modifications of PLGA can promote its adhesion to cells and their extracellular matrix (ECM). Bioactive glasses are attractive modifiers of PLGA due to their strong bone-binding ability as well as their own potential to stimulate stem cells to undergo osteogenesis [7]. The first bioactive glass (BG), discovered by Hench et al. in 1971, composed of 45SiO_2_–24.5Na_2_O–24.5CaO–6P_2_O_5_ (wt%) and referred to as 45S5 Bioglass^®^ [8], has been commercialized for broad clinical use in restorative dentistry, craniofacial surgery, and orthopedics due to its strong bone-binding capabilities [9, 10]. Studies investigating the osteogenic responses of ASCs to 45S5-based scaffolds have shown increased cellular alkaline phosphatase (ALP) activity [11] and collagen type I synthesis [12] in rather long-term 5-6-week ASC cultures. Other modifications of bioactive glasses showed the potential to induce the expression of early osteoblastic markers such as runt related-transcription factor 2 (*RUNX2*) and osterix (*OSX*) and change cell attachment-related intracellular signaling pathways of ERK1/2, FAK and JNK in approximately 2-3-week ASC cultures [13].

Due to the limited structural resistance of bioactive glasses, the various polymer-based composites are extensively explored. Recently, we have shown that PLGA-based composites modified with SiO_2_-CaO or SiO_2_-CaO-P_2_O_5_ sol-gel-derived bioactive glasses (SBGs) stimulate BMP-dependent intracellular signaling pathways and enhance osteogenic marker expression in standard human BMSC cultures, i.e., without medium supplementation with osteogenic growth factors [14]. In the present study, we applied the abovementioned SBG-PLGA composites to support human ASC osteogenesis. However, given that ASCs display weaker osteogenic potential than BMSCs [4], we introduced SBG modifications with strontium or zinc oxides, as well as specific culture medium supplementation and dynamic culture conditions.

Bone morphogenetic proteins (BMPs) play a pivotal role in skeletal development and remain the most studied in vitro and in vivo for their osteogenic properties [15]. Among the 20 members of the BMP family, recombinant human BMP-2 has been approved for restricted use in spinal fusion [16]. However, due to the supraphysiological doses of rhBMP-2 (1.5 mg/ml) present in the latter medical device, the off-label applications have been reported to result in several side effects such as inflammation and hematoma formation [17, 18]. We have also shown that stimulation of ASCs with rhBMP-2 (100 ng/ml) alone is not sufficient for osteogenic differentiation [19], but rhBMP-2 has been widely shown to be useful in different investigated settings. Since bone morphogenetic proteins (BMPs) display osteoinductive properties in MSC cultures synergistically with bioactive glasses [20] and/or under dynamic cell culture conditions [21], we have based the culture medium supplementation on BMP signal amplification. BMP signaling has been implicated in the induction of ASC osteogenesis [22]. BMPs transmit signals to cells through both canonical and noncanonical pathways, the former involving SMAD proteins and the latter involving ERK, PI3K, and JNK kinases [23]. BMP signaling can be amplified e.g., by Phenamil that results in the Trb3 protein production, which inhibits Smurf1-mediated degradation of SMAD1/5/8 proteins involved in canonical BMP signal transduction [24]. Osyczka and Leboy have also shown that BMP-2-mediated osteogenesis of human BMSCs can be enhanced by ERK pathway inhibition [25].

Mechanical stimulation (stretching, compression, fluid shear stress, etc.) is necessary for bone homeostasis as bone tissue responds to mechanical loading by adjusting its structure and density. MSCs can convert mechanical stimuli by e.g., primary cilia, mechanosensitive ion channels and integrins [26]. It has been demonstrated that fluid shear stress can promote the osteogenic differentiation of MSCs [27, 28], osteoblasts and modify the network of osteocytes [29]. The perfusion of culture media applied to ASCs cultured on decellularized bone scaffolds has been proven to increase the secretion of bone extracellular matrix components [30]. Furthermore, when ASCs were cultured on BG-based scaffolds, media perfusion acted synergistically with the properties of BGs to enhance the expression of osteogenic markers such as osteocalcin and osteopontin [31].

Among other factors facilitating osteogenesis, zinc and strontium have been shown to contribute to bone homeostasis, besides their antibacterial and anticancer properties [32–34]. Zinc participates in various biological processes as it serves as a cofactor of many enzymes and transcription factors. In skeletal tissue zinc has been shown to accumulate in developing osteons and bone apatite [35]. Zinc promotes the differentiation of murine osteoblastic cells by upregulating the expression of the transcription factor *RUNX2* via BMP-related SMAD1 phosphorylation [36]. In addition, zinc not only is essential for ALP enzyme activity, collagen synthesis and ECM mineralization by osteoblasts, but also it prevents bone resorption by facilitating the RANKL/RANK/OPG axis as zinc deficiency has been observed in osteoporotic patients [37, 38]. Zinc has been successfully incorporated into BGs [39], resulting in increased mineralization in rat BMSCs [40] and in human osteoblastic SaOS-2 cells [41]. Similar approaches involving strontium-doped BGs have been investigated as strontium ranelate has been clinically used as an anti-osteoporotic agent [42]. Strontium is a trace element that acts similarly to Ca^2+^ and is mainly deposited in bone [43]. When incorporated into BGs, Sr-BG nanoparticles stimulated early and late osteoblastic gene expression in human BMSCs [44]. Moreover, Sr-BGs reportedly may prevent bone resorption by inhibiting RANKL-mediated osteoclastogenesis and reducing TRAP activity in murine macrophage cells [45, 46].

This work presents novel strategies for effective osteogenic differentiation of human ASCs by combining culture on bioactive composite growth surfaces, treatment of cells with specific chemical compounds added to osteogenic culture medium, and application of dynamic culture conditions by gentle cell culture rocking. We show that the treatment of ASCs with Phenamil and a MEK1/2 inhibitor effectively maintains BMP-2-stimulated osteogenesis in long-term ASC cultures on PLGA-based composite sheets containing 50 wt% sol-gel bioactive glasses from the SiO_2_-CaO±P_2_O_5_ system. Furthermore, the enrichment of the SiO_2_-CaO±P_2_O_5_ with 5 wt% SrO or ZnO in PLGA-based composites, combined with fluid shear stress and the abovementioned medium supplements, results in the rapid expression of bone matrix-related markers in 3-day cultures and robust matrix mineralization by day 12 of culture, following a short, 7-day ASC preculture on bioactive composites. We also determined that the applied osteogenic strategy contributes to the activation of β-catenin and CREB or COX-2 expression. Autologous administration of undifferentiated ASCs has been evaluated in clinical trials mainly for knee osteoarthritis and cartilage tissue regeneration [47]. However, ASC-derived differentiated cell phenotypes have not yet been used in bone tissue therapies due to their long differentiation time in vitro. Here, we show the novel approaches to rapidly and effectively obtain ASC-derived bone cells for potential future administration to fractured or diseased bone sites, e.g., during restorative dentistry, maxillofacial or orthopedic surgeries.

## Results

### Human ASC culture on SBG-PLGA composites supports rhBMP-2 mediated osteogenesis

**Fig. 1:**
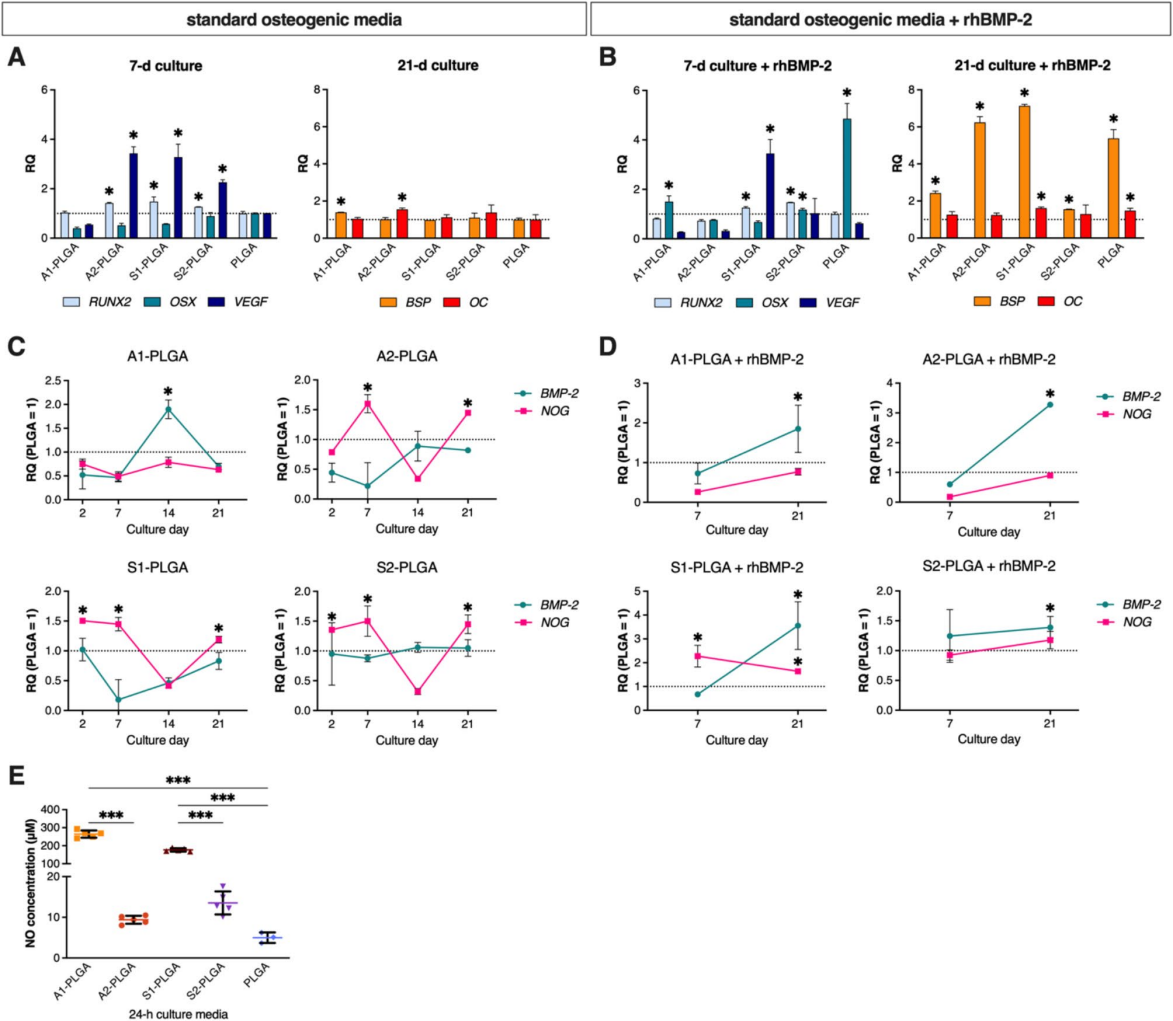
Human ASC osteogenesis is supported by culture on SBG-PLGA composites and rhBMP-2 treatment. mRNA levels of the early and late osteoblastic markers, *BMP-2* and Noggin (*NOG*) in human ASCs cultured on SBG-PLGA composites in (**A**), (**C**) standard osteogenic medium or (**B**), (**D**) standard osteogenic medium supplemented with 100 ng/ml rhBMP-2. Results are presented as relative mRNA expression levels vs. mRNA levels for ASCs cultured on a plain PLGA control (black line at 1). (**E**) Nitric oxide (NO) concentration in culture media after 24-h culture of ASC cells on SBG-PLGA composites in standard osteogenic medium. Averages ± SD are indicated. One-way or two-way ANOVA tests, *p < 0.05, **p < 0.001, ***p < 0.0001 relative to the PLGA control group

We used previously described by us [14, 48] PLGA-based, SBG-enriched composites (Fig. 6) to assess their potential to support the osteogenesis of human adipose-derived stem cells (ASCs; ASC52telo, ATCC). Initially, ASCs were cultured in standard osteogenic medium consisting of ascorbic acid, dexamethasone and beta-glycerophosphate. To determine potential differences in ASC response depending on the composition of the bioactive glass, four different SBGs were used as modifiers of PLGA, including high calcium (A type) and high silica (S type) of CaO-SiO_2_ (type 1) and CaO-SiO_2_-P_2_O_5_ (type 2) systems (see Table 1 for SBG composition) vs. the control PLGA surface. The osteogenic potential of these composites had been previously extensively studied in human BMSCs without any other osteogenic treatments [48]. In the present study, ASCs were analyzed for the expression of either early osteogenic transcription factors/cytokines or for late osteoblastic markers associated with bone matrix. On day 7 of culture, we detected increased mRNA levels of osterix (*OSX*), one of a key transcription factor driving osteogenesis [49], and increased mRNA levels of vascular endothelial growth factor (*VEGF*), a key cytokine involved in angiogenesis [50], in ASCs grown on A2, S1 and S2-enriched PLGA composites. However, in longer cultures (i.e., up to day 21), the mRNA expressions of typical bone-related markers, i.e., bone sialoprotein (*BSP*) and osteocalcin (*OC*), were not increased on most of the studied composites, except for A1-PLGA and A2-PLGA, respectively (Fig. 1a). We have thus assumed that the osteogenesis of ASCs is initiated, but not maintained by the abovementioned culture set-up. To investigate the potential reasons for the lack of ASC osteogenic progression, we examined the mRNA expression of BMP-2 along with its inhibitor Noggin at different human ASC culture time points. Overall, on day 7 of culture the expression of Noggin was higher than the expression of BMP-2 in ASCs cultured on the A2-, S1- and S2-PLGA composites. In longer cultures, Noggin expression decreased by day 14, but increased again by day 21 of culture (Fig. 1c) on all composites, except A1-PLGA. Given that the studied composites can induce BMP expression and BMP-related osteogenic signaling in human BMSC cultures [14], the second increase (day 21) in Noggin expression in longer ASC cultures may have prevented the progression of osteogenesis. We also noted that the osteogenic response of ASCs was modest for the A1-PLGA composites (despite the increased expression of BMP-2 at day 14), which were previously shown to release the highest amounts of calcium ions [14]. To overcome the latter and support ASC osteogenesis in long-term cultures on SBG-PLGA composites, we supplemented standard osteogenic medium with recombinant human BMP-2 (rhBMP-2, 100 ng/ml). The addition of exogenous rhBMP-2 to human ASCs cultured on SBG-PLGA composites resulted in lower *NOG* mRNA expression, increased *BMP-2* mRNA expression and enhanced mRNA expression of both early and late osteogenic markers (Fig. 1b, d).

Since calcium contributes to nitric oxide (NO) production [51] and excess NO can inhibit the growth of osteoblasts [52], we determined that ASCs cultured on both A1- and S1-PLGA composites (i.e., those depleted of P_2_O_5_) produced significantly higher amounts of NO (150–250 µM), compared to the A2- and S2-PLGA composites (5–20 µM) (Fig. 1e). This is consistent with the overall higher release of calcium ions by SBG-PLGA composites depleted of P_2_O_5_ [14]. Considering that the highest NO levels (250 µM) were detected for ASCs cultured on A1-PLGA composites, this was plausible reason for their poorest osteogenic response.

### Phenamil and PD98059 enhance rhBMP-2-mediated early and late osteogenic mRNA expression in ASCs cultured on SBG-PLGA composites

**Fig. 2:**
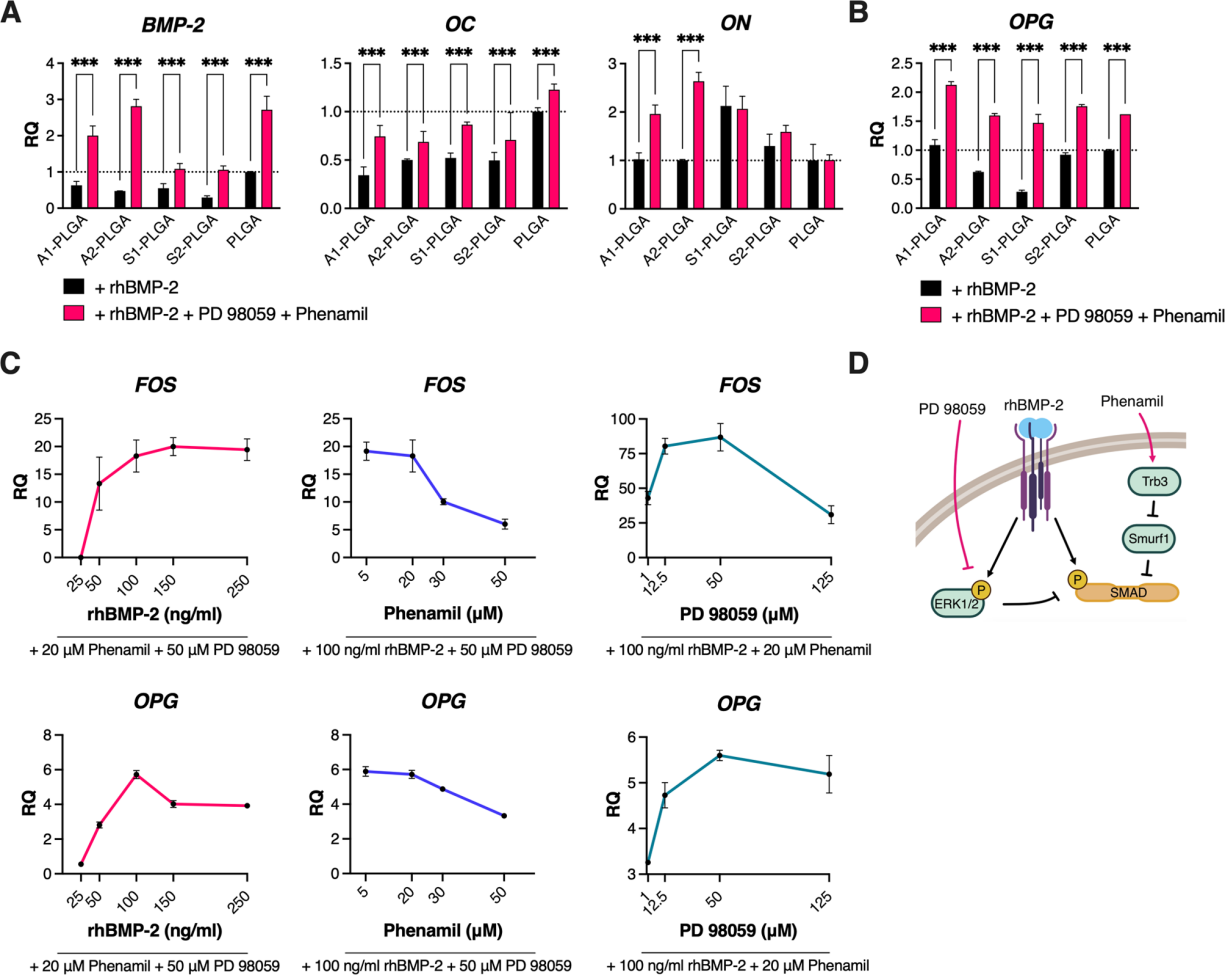
Cumulative osteogenic effect of Phenamil and PD98059 treatment in rhBMP-2 stimulated human ASCs cultured on SBG-PLGA composites. mRNA levels of osteoblastic markers in (**A**) 7-day and (**B**) 21-day ASC cultures on SBG-PLGA composites. ASCs were cultured in osteogenic medium supplemented with 100 ng/ml rhBMP-2 or 100 ng/ml rhBMP-2, 50 µM PD98059 and 20 µM Phenamil. Results are presented as relative mRNA expression compared to mRNA levels in control cells cultured on PLGA with rhBMP-2 only (marked as black line at 1). (**C**) mRNA levels of selected osteoblastic markers in 3-day osteogenic ASC cultures treated with different doses of rhBMP-2 (25–250 ng/ml), Phenamil (5–50 µM) or PD98059 (1-125 µM); under fluid shear stress. Results are presented as the expression relative to osteogenic cultures treated only with ascorbic acid, dexamethasone and β-glycerophosphate. (**D**) Graphical hypothesis of BMP-2, PD98059 and Phenamil cross-talk in intracellular signaling. Average values ± SD are indicated. Two-way ANOVA test, *p < 0.05, **p < 0.001, ***p < 0.0001 relative to the PLGA control group or between marked groups. *BMP-2* – bone morphogenetic protein 2, *OC* – osteocalcin, *ON* – osteonectin, *FOS* – AP-1 transcription factor subunit (c-fos), *OPG* – osteoprotegerin

Since the addition of rhBMP-2 to osteogenic ASC cultures on SBG-PLGA composites improved their osteogenic progression, we sought to enhance canonical BMP-2 signaling by adding the MEK1/2 kinase inhibitor PD98059 (50 µM) and the indirect Smurf1 inhibitor Phenamil (20 µM) along with rhBMP-2 to standard osteogenic medium. We assessed the expression of selected osteogenic markers in 7-day ASC cultures treated with rhBMP-2 and either PD98059 or Phenamil or both. These data showed that rhBMP-2 treatment with both PD98059 and Phenamil resulted in higher expression of *BMP-2* and osteocalcin (*OC*) compared to PD98059 or Phenamil added separately with rhBMP-2 (Supplementary Fig. 1). After 7 days of such combined ASC treatment, we detected significant increases in *BMP-2* and *OC* mRNA levels in all tested cell cultures vs. cultures treated solely with rhBMP-2 (Fig. 2a). Furthermore, combined ASC treatment resulted in significantly increased osteonectin (*ON*) mRNA levels in cells cultured on A1- and A2-PLGA (Fig. 2a). After 21 days of combined cell treatment, osteoprotegerin (*OPG*) mRNA was elevated in all cells grown on the tested composites vs. respective cultures treated with rhBMP-2 only (Fig. 2b). To determine the optimal doses of each component of the aforementioned chemical cocktail, ASCs were treated with rhBMP-2 (25–250 ng/ml), PD98059 (1-125 µM), and Phenamil (5–50 µM). When the concentration of one compound varied, the concentrations of the remaining compounds were set at initial values (see Fig. 2c). It was observed that the highest expression of c-fos (*FOS*, an immediate osteogenic stimulus-responding gene [53]) and osteoprotegerin (*OPG*) was achieved with a combination of 100 ng/ml rhBMP-2, 50 µM PD98059 and 20 µM Phenamil (Fig. 2c). Therefore, it can be concluded that each component (at the initial doses) is necessary to obtain the most effective osteogenic response in ASCs.

### The application of fluid shear stress in ASCs cultured on SBG-PLGA composites promotes expression of early osteogenic markers and strengthens osteogenic effects of rhBMP-2, PD98059 and Phenamil

**Fig. 3:**
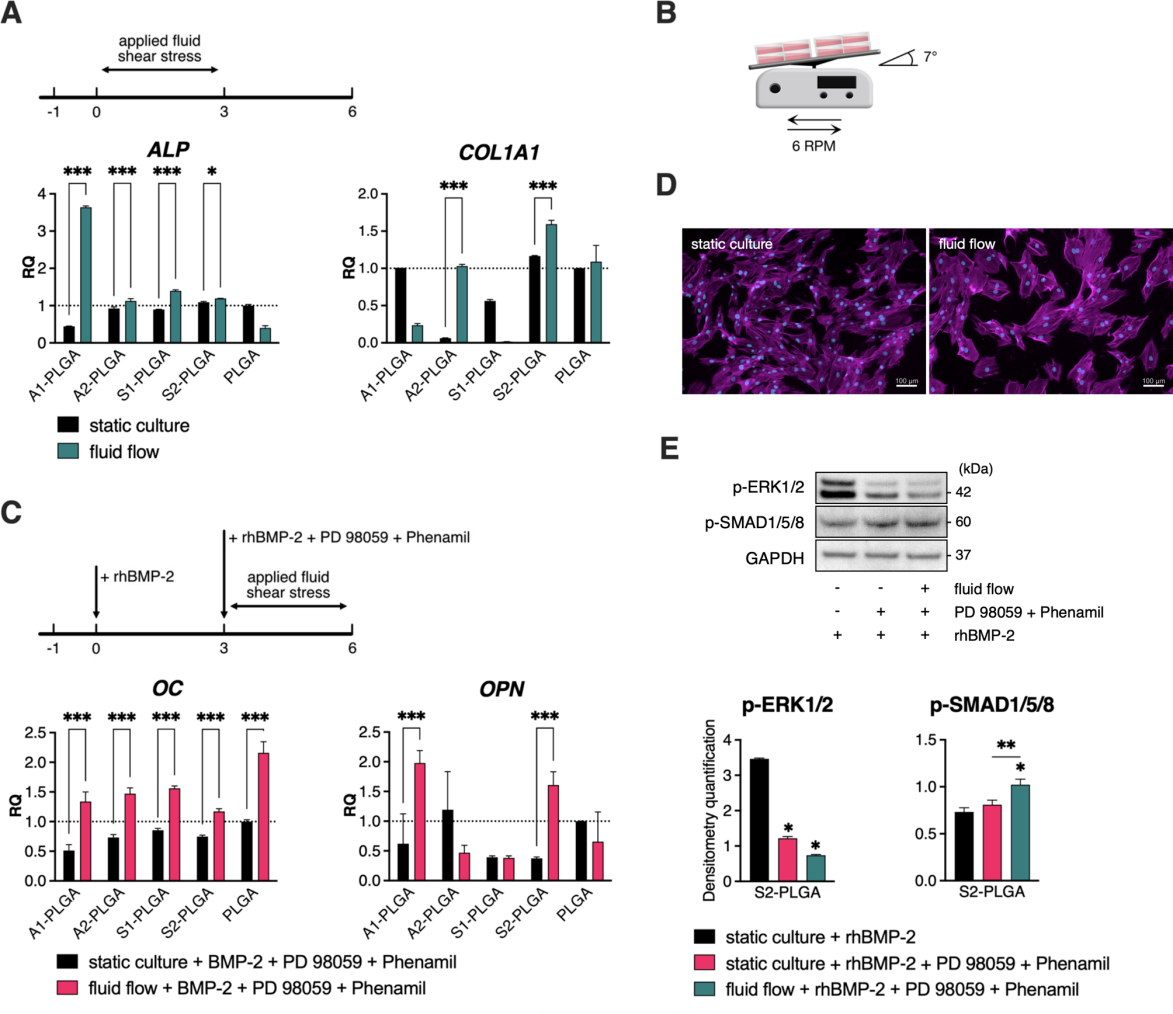
Fluid shear stress strengthens the osteogenic effects of rhBMP-2, PD98059 and Phenamil in ASCs cultured on SBG-PLGA composites. mRNA levels of osteoblastic markers after 7-day ASC culture on SBG-PLGA composites in (**A**) standard osteogenic medium under either static conditions or with fluid shear stress; and (**C**) osteogenic medium supplemented with 100 ng/ml rhBMP-2, 50 µM PD98059 and 20 µM Phenamil under either static conditions or with fluid shear stress. Results are presented as relative mRNA expression levels compared to mRNA levels in a control, static culture on PLGA (marked as a black line at 1). (**B**) The method of fluid shear stress application in ASC cultures using a standard laboratory see-saw rocker (7° tilt angle, 6 RPM frequency). (**D**) F-actin distribution in ASCs (Phalloidin-Atto488, magenta colored) at day 3 of culture in osteogenic medium supplemented with rhBMP-2, PD98059 and Phenamil after continuous static or dynamic culture conditions applied for 3 days. Scale bar represents 100 μm. (**E**) Western blot (WB) analysis of p-ERK1/2 and p-SMAD1/5/8 in ASCs after 1-h treatment with rhBMP-2 or rhBMP-2, PD98059 and Phenamil in static or dynamic conditions (upper panel) along with densitometric quantifications of WB results normalized to GAPDH levels. Averages ± SD are indicated. Two-way ANOVA test, *p < 0.05, **p < 0.001, ***p < 0.0001 relative to the static PLGA control or between marked groups

We have introduced fluid shear stress by gentle, horizontal rocking of established cell cultures using a standard laboratory see-saw rocker (Fig. 3) within a 7-day cell culture time frame. The applied low-magnitude fluid shear stress of approx. 2 mPa is typical for see-saw rocking of cell cultures and it can be increased by either lowering the cell culture media volume, increasing the tilt angle or shortening the cycle time. In this work the fluid shear stress was calculated using the equation:

​​$$\:{\uptau\:}=\frac{\pi\:\mu\:{\theta\:}_{max}}{{2\left(\frac{{h}_{0}}{L}\right)}^{2}T}$$ [54],

where $\:\mu\:$ is the fluid viscosity, $\:{\theta\:}_{max}$ the maximal flip angle, $\:{h}_{0}$ the fluid depth, *L* the well length and *T* the time for one cycle. The fluid shear stress applied in this study is relatively low, but it can be easily introduced to enhance the osteogenic differentiation of ASCs in any preclinical setting. However, the higher fluid shear stress at physiological rates (0.001–3 Pa) [29] can be expected to contribute even more effectively to osteogenic differentiation (e.g., at 0.5 Pa [55]). Cells were cultured on SBG-PLGA composites in either standard osteogenic medium or osteogenic medium supplemented with BMP-2, PD98059 and Phenamil. In our previous study, we demonstrated that even a single 2-h session of media perfusion induces osteogenesis in hBMSCs cultured in 3D scaffolds [56], and in 2D cultures Yourek et al. showed that 24 h of exposure to fluid shear stress induces hBMSC osteogenesis [27]. First, we assessed the impact of continuous low-magnitude fluid shear stress that was applied in the first 3 days of 7-day ASC cultures on SBG-PLGA composites in standard osteogenic medium. The application of fluid flow on days 0–3 of the 7-day cultures that were maintained in osteogenic medium resulted in increased alkaline phosphatase (*ALP*) mRNA levels on all studied composites and increased collagen type I (*COL1A1*) mRNA on the A2- and S2-PLGA composites (Fig. 3a). Further, we aimed to combine the observed positive effects of rhBMP-2, Phenamil and PD98059 treatment (Fig. 2) with 3-day application of fluid shear stress. Cultures grown in osteogenic medium supplemented with rhBMP-2, PD98059 and Phenamil and stimulated with fluid flow at days 3–6 displayed increased osteocalcin (*OC*) mRNA levels on all studied composite surfaces and increased osteopontin (*OPN*) mRNA levels on the A1- and S2-PLGA composites (Fig. 3c). Thus, the application of fluid flow enhanced the osteogenic effect of SBG-PLGA composite surfaces as well as the combined cell treatment by rhBMP-2, PD98059 and Phenamil. In addition, we observed the changes in F-actin cytoskeleton and enlarged cell shape after 3 days of continuous fluid shear stress compared to static culture (Fig. 3d). We also assessed the phosphorylation levels of ERK1/2 and SMAD1/5/8 proteins in ASCs cultured on S2-PLGA composites and treated for 1 h with rhBMP-2 (under static conditions) or rhBMP-2, PD98059, Phenamil (under static or dynamic conditions). Western blot analysis showed that PD98059 decreased ERK1/2 phosphorylation in both static and dynamic cultures, while fluid shear stress application increased p-SMAD1/5/8 in ASCs treated with rhBMP-2, PD98059 and Phenamil (Fig. 3e).

### Modification of SBGs with ZnO or SrO in PLGA-based composites strengthens the osteogenic effects of combined ASC treatment as well as the effects of fluid shear stress

**Fig. 4:**
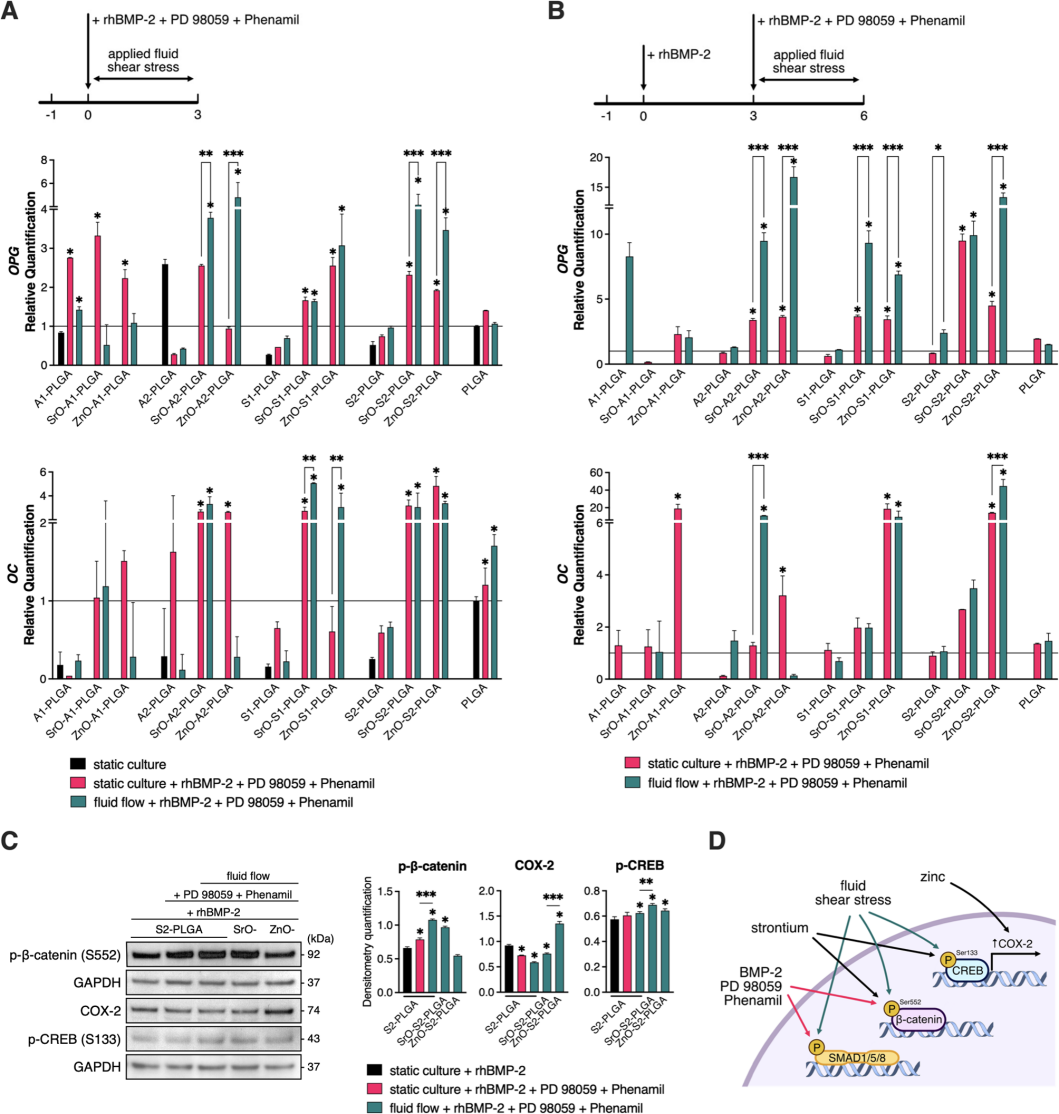
Zinc (ZnO) or strontium (SrO) modified SBG-PLGA composites combined with fluid shear stress and BMP-based chemical stimulation, further increase osteogenic markers expression in early ASC cultures. mRNA levels of osteoblastic markers in (**A**) 3-day and (**B**) 6-day osteogenic ASC cultures on PLGA-based composites containing either unmodified or SrO- or ZnO-modified SBGs. Cells were treated with a combination of rhBMP-2, PD98059 and Phenamil at the indicated culture times in either static cultures or under fluid shear stress. Upper panels show the schemes of the ASC treatments. Results are presented as relative mRNA expression levels vs. mRNA levels in a control, static culture on PLGA (marked as a black line at 1). (**C**) Western blot (WB) analysis of phospho-β-catenin(Ser552), COX-2 and phospho-CREB(Ser133) levels in ASCs after 1-h treatment with rhBMP-2 or rhBMP-2, PD98059 and Phenamil in static or dynamic conditions (left panel) along with densitometric quantifications of WB results normalized to GAPDH levels (right panel). (**D**) Hypothesized signaling pathways involved in osteogenic response to treatment strategy. Averages ± SD are indicated. Two-way ANOVA test, *p < 0.05, **p < 0.001, ***p < 0.0001 relative to the respective static PLGA control group or between marked groups

Given that zinc and strontium ions can positively influence osteogenesis [32, 34], we modified the studied SBGs [48] with 5 wt% of either zinc oxide (ZnO) or strontium oxide (SrO) [57] (for details, see Table 1). The obtained PLGA-based composites containing either modified or unmodified SBGs were used as growth surfaces for ASC cultures. The cells were stimulated with rhBMP-2, PD98059 and Phenamil in either static cultures or fluid shear stress was applied at a given culture time frame. Specifically, we verified the efficacy of the combined osteoinduction strategy (chemical stimulation and fluid shear stress) applied from the beginning of the 3-day cultures (Fig. 4a), as well as the pretreatment with rhBMP-2 to direct ASCs to the osteogenic lineage before applying the osteoinduction strategy on day 3 in 7-day cultures (Fig. 4b). Our results showed that 3-day static ASC cultures on the composites containing modified SBGs along with cells treated with rhBMP-2, PD98059, and Phenamil resulted in increased mRNA levels of osteoprotegerin (*OPG)* and osteocalcin (*OC*) (Fig. 4a, pink bars) on all examined composites except for SrO- or ZnO-modified A1-PLGA composites vs. the cultures on the composites with unmodified SBGs. The introduction of fluid flow to the abovementioned cultures further increased *OPG* mRNA levels in ASCs cultured on ZnO- and SrO-modified A2- and S2-PLGA composites, and *OC* mRNA levels in those cultured on SrO- or ZnO-modified S1-PLGA (Fig. 4a, green vs. pink bars). The modification of the abovementioned culture scheme by a 3-day ASC treatment with BMP-2 followed by a 3-day combined cell treatment along with the application of fluid shear stress at days 4–7 led to significant increases in *OPG* mRNA levels in ASCs cultured on ZnO- or SrO-modified A2-, S1- and S2-PLGA composites (Fig. 4b, left panel). This culture scheme also resulted in significant increases in *OC* mRNA levels in ASCs cultured on SrO-modified A2-PLGA or ZnO-modified S2-PLGA (Fig. 4b, right panel). Since the results shown in this study were obtained using ASC52telo cell line, we verified that in normal human primary ASCs the combined chemical treatment with fluid flow upon 3-day culture on ZnO- or SrO-modified S2-PLGA also resulted in increased mRNA expression of osteopontin (*OPN*), *OC* and *OPG* (Supplementary Fig. 2).

Although the molecular mechanism, by which zinc or strontium incorporation into SBG-PLGA composites, together with BMP-based chemical stimulation and applied fluid shear stress induce rapid ASC osteogenic response were not the focus of this study, we have investigated the plausible signaling pathways that may be activated under such combined treatment. ASCs cultured on unmodified S2-PLGA or SrO-/ZnO-modified composites were treated for 1 h with rhBMP-2 (under static conditions) or rhBMP-2, PD98059, Phenamil (under static or dynamic conditions). Western blot analyses showed that PD98059 and Phenamil led to increased phosphorylation of β-catenin at Ser552 (promoting its transcriptional activity) and fluid shear stress even further enhanced its phosphorylation levels on S2-PLGA and SrO-S2-PLGA composites. We also observed that fluid shear stress elevated the phosphorylation of CREB, with the highest level in ASC culture on SrO-S2-PLGA. While ASC culture on ZnO-S2-PLGA together with culture media flow enhanced the expression of COX-2 (Fig. 4c).

**Fig. 5:**
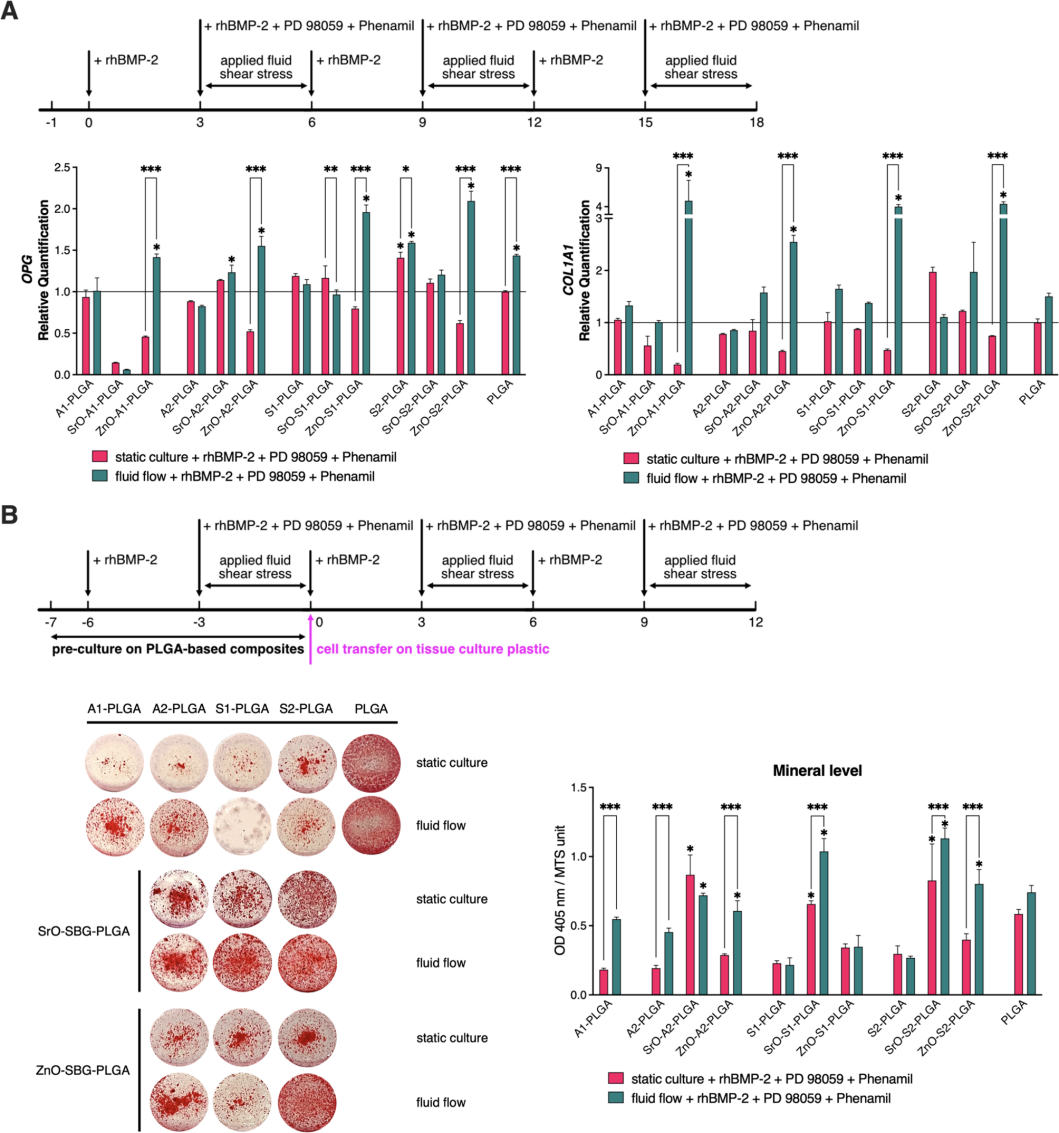
A short ASC preculture on ZnO- or SrO-modified SBG-PLGA growth surfaces, combined with BMP-based chemical treatment and fluid shear stress, leads to robust mineral deposition in further cultures on typical tissue culture plates. (**A**) The mRNA levels of *OPG* and *COL1A1* in 18-day ASC cultures on PLGA-based composites containing either unmodified or SrO- or ZnO-modified SBGs. Cells were treated with 100 ng/ml rhBMP-2, 50 µM PD98059 and 20 µM Phenamil in either static conditions or with fluid shear stress. The upper panel shows the scheme of ASC treatment. Results are presented as relative mRNA expression vs. mRNA levels in a control, static culture on PLGA (marked as a black line at 1). (**B**) Mineral levels (Alizarin Red S staining) in ASC cultures after 7-day preculture on the indicated composites followed by 12-day culture in standard tissue culture plates (left panel). The upper panel shows the scheme of ASC treatment. Colorimetric quantification of Alizarin Red S staining normalized to the number of live cells (right panel). Averages ± SD are indicated. Two-way ANOVA test, *p < 0.05, **p < 0.001, ***p < 0.0001 relative to the PLGA static control

We have also extended the culture scheme shown in Fig. 4b to apply it repeatedly 3 times in ASC cultures on PLGA-based composites containing either unmodified or SrO- or ZnO-modified SBGs (Fig. 5a). Cells were treated with a combination of rhBMP-2, PD98059 and Phenamil at the indicated culture times under either static conditions or fluid shear stress. On culture day 18, combined cell treatment along with the application of fluid flow led to increased *OPG* and *COL1A1* mRNA levels on all composites containing ZnO-modified SBGs and increased *COL1A1* mRNA levels on composites containing SrO-modified A2 or S2 SBGs. Notably, this culture scheme also resulted in increased *OPG* and *COL1A1* mRNA levels in ASCs cultured in the PLGA control. Furthermore, we precultured cells on the selected composites for 7 days under the abovementioned conditions, followed by cells transfer to standard cell culture plates (i.e., without bioactive composites) and continuous cell treatment with the same scheme (Fig. 5b). This resulted in significant amounts of mineralized extracellular matrix being produced by ASC cells. The highest mineral levels were produced by ASCs precultured on composites containing SrO-modified S1 and S2 SBGs and ZnO-modified S2 SBG when both combined cell treatment and fluid flow were applied to the preculture. Whereas preculturing cells on the composites under static culture conditions, resulted in the highest mineral levels for cells precultured on composites containing SrO-modified A2, S1 and S2 SBGs. Overall, these findings indicate that ASCs can be efficiently committed toward mineralizing osteoblasts after short-term preculture on composites containing SrO- or ZnO-modified SBGs and continuous cell treatment with a combination of rhBMP-2, PD98059, and Phenamil.

## Discussion

In this study, we sought to develop a rapid and effective osteoinduction strategy for human ASCs upon their culture on PLGA-based composite surfaces containing 50 wt% sol-gel bioactive glasses (SBGs) of the SiO_2_-CaO ± P_2_O_5_ system. We used either high calcium (A1, A2) or high silica (S1, S2) SBGs to determine whether they are equally suitable to support human ASC osteogenesis (Table 1). Previously, we determined that such composite growth surfaces displayed osteoinductive properties in human bone marrow-derived stromal cell (BMSC) cultures and promoted BMSCs to osteogenesis on their own, without other osteogenic culture supplements [14, 58, 59]. Given the above, it was plausible to use such composite growth surfaces for osteogenic differentiation of human ASCs, especially that they may serve as human ASC delivery vehicles. Since the osteogenic potential of ASCs is reportedly lower than that of BMSCs, we supplemented standard osteogenic culture medium with recombinant human BMP-2 (rhBMP-2), the MEK1/2 inhibitor (PD98059) and Phenamil. ASCs were grown on the aforementioned SBG-PLGA composites, either unmodified or modified with SrO or ZnO, under static or dynamic culture conditions (Figs. 3, 4 and 5).

The applied SiO_2_-CaO ± P_2_O_5_-PLGA composites were previously shown to activate endogenous BMP expression and signaling in human BMSCs followed by BMSC osteogenic progression [14, 58, 59]. Our present study shows that human ASC cultures can undergo osteogenic differentiation upon their growth on the abovementioned composites in standard osteogenic medium containing ascorbic acid, dexamethasone, and beta-glycerophosphate, but their osteogenesis does not progress in longer cultures (Fig. 1a). We hypothesized that this may be related to either low endogenous BMP expression or increased expression of the BMP inhibitor – Noggin in longer cultures. Indeed, ASCs cultured on the studied composites in standard osteogenic medium (i.e., all except A1-PLGA) did not show a substantial increase in endogenous BMP-2 expression and the Noggin expression levels were overall higher than those of endogenous BMP-2 (Fig. 1c). Some authors reported that silencing of Noggin expression along with exogenous BMP-2 stimulation in ASC cultures that resulted in increased ASC osteogenic differentiation on chitosan, PCL or PLGA scaffolds [20, 60, 61]. However, we show that continuous ASC treatment with rhBMP-2 in long-term culture on SBG-PLGA composites changes the overall mRNA ratio of Noggin (*NOG*) to endogenous *BMP-2* and significantly increases bone sialoprotein (*BSP*) and osteocalcin *(OC)* mRNA expression (Fig. 1b, d). Thus, ASC stimulation with rhBMP-2 improves their osteogenic progression upon culture on SBG-PLGA composites. However, we show that continuous treatment of ASCs with rhBMP-2 in long-term culture on SBG-PLGA composites alters the overall mRNA ratio of Noggin (NOG) to endogenous BMP-2 and significantly increases bone sialoprotein (BSP) and osteocalcin (OC) mRNA expression (Fig. 1b, d). Thus, stimulation of ASCs with rhBMP-2 improves their osteogenic progression when cultured on SBG-PLGA composites. However, our results also indicate that culturing human ASCs on the composites containing bioactive glasses without P_2_O_5_ results in higher NO production by ASC cells (Fig. 1e). This may be due to a higher calcium release rate by such composites [14]. The latter may stimulate NO production to the levels inhibitory for osteoblast growth [51, 62], despite e.g. A1-PLGA surfaces promoted high expression of endogenous BMP-2 (Fig. 1c). Therefore, the composites containing bioactive glasses without P_2_O_5_ may present some limitations for human ASC osteogenic cultures.

Given that human ASCs cultured on SBG-PLGA and treated with rhBMP-2 showed decreased Noggin expression vs. endogenous BMP-2, we aimed to enhance the osteogenic action of BMP-2 with PD98059 and Phenamil. Osyczka & Leboy showed that the addition of the MEK1/2 inhibitor (PD98059) to human BMSC cultures enhanced the osteogenic action of rhBMP-2 [25]. More recently, Fan et al. reported enhanced mineralization in murine ASCs after 3-week culture treatment with BMP-2 and Phenamil and increased ALP activity after 2-week culture on PLGA scaffolds soaked with 10 µg/ml BMP-2 and 200 µM Phenamil [24]. A direct comparison of our studies to those of Fan et al. is problematic as the latter authors used murine ASC cells that are known to differentiate faster than human cells [63] and the authors soaked PLGA scaffolds with much higher doses of the above agents [24]. In our studies, the stimulation of human ASCs with combined medium supplements (i.e., rhBMP-2, PD98059 and Phenamil) upon culture on SBG-PLGA composites markedly increased the expression of endogenous BMP-2, as well as osteonectin and osteocalcin after 7-day culture and osteoprotegerin after 21-day culture (Fig. 2a, b). Our results also demonstrate that the treatment with a lower/higher dose of any chemical cocktail component or treatment with either PD98059 or Phenamil separately is less effective in inducing the osteogenic response of ASCs than the doses initially established by us for combined rhBMP-2, PD98059 and Phenamil treatment (Fig. 2c, Supplementary Fig. 1). Following the studies of Fan et al. [24], we hypothesized that Phenamil could stabilize BMP-related SMAD1/5/8 intracellular signal transducers by activating the tribble homolog 3 (Trb3) protein and thus blocking Smad ubiquitin regulatory factor (Smurf1) [24]. Whereas the addition of PD98059 could have resulted in ERK inhibition and thus prevented the phosphorylation of SMAD1/5/8 linker regions and SMAD inhibition/degradation [64, 65]. Indeed, our chemical cocktail decreased phosphorylation of ERK1/2 and slightly increased p-SMAD1/5/8 levels in ASC cultures (Fig. 3e). Taken together, we show that culture of human ASCs on SBG-PLGA growth surfaces along with their treatment with rhBMP-2, Phenamil and PD98059, markedly enhance the expression of osteogenic markers in human ASCs. We do not intend to use our chemical cocktail in vivo, but it is worth noting that compared to clinically approved delivery of rhBMP-2 at the 1.5 mg/ml concentration [16], our co-treatment with Phenamil and PD98059 made the dose of 100 ng/ml rhBMP-2 effective in ASC osteoinduction, although in our previous work the treatment with only 100 ng/ml rhBMP-2 was not sufficient to stimulate osteogenesis in ASCs [19].

Various mechanical stimuli such as shear forces, compression, pressure, or tension modulate bone formation and regeneration [21]. The shear forces generated by fluid flow in the canaliculi system are a type of mechanical signal received by osteocytes [21]. It has also been shown that fluid shear stress applied to MSC cultures promotes osteogenic differentiation [21, 27–29, 66]. Particularly, in long-term human and rat ASC cultures on different (collagen or decellularized bone) scaffolds, fluid shear stress, applied by culture medium perfusion, increased extracellular matrix mineralization [30, 67]. Mechanotransduction has also been linked to BMP signaling pathways [21, 68] e.g., by increasing the sensitivity of osteoblasts to BMP-2 through the activation of SMADs as the immediate cell response or by influencing BMP-related vascularization processes in blood vessels [69].Thus, we decided to introduce fluid shear stress to ASC cultures using a standard laboratory rocker, at 3-day culture intervals (Fig. 3c). Notably, after 1 h of a fluid flow application and chemical treatment, we observed the elevated phosphorylation of SMAD1/5/8 in ASC cultures on S2-PLGA composites, compared to cells in static conditions (Fig. 3e). The application of fluid shear stress to 7-d ASCs cultured on SBG-PLGA composites in standard osteogenic medium enhanced the expression of alkaline phosphatase (ASCs on all studied composites) and collagen type I (ASCs on A2- and S2-PLGA composites, Fig. 3a). Similar synergistic effects of media perfusion and bioactive glass surfaces/scaffolds in human ASC osteogenic cultures were observed by Silva et al. [31]. Whereas, in our studies, treatment with BMP-2, Phenamil, and PD98059 combined with fluid shear stress (Fig. 3c) resulted in increased osteocalcin (all studied composites as well as control PLGA) and osteopontin (A1- and S2-PLGA) mRNA levels in human 7-d ASC cultures. This suggests that fluid shear stress acts synergistically with medium supplements (i.e., rhBMP2, Phenamil and PD98059) and enhances the osteogenic response of human ASCs cultured on the SBG-PLGA composites.

Since Zn and Sr ions are emerging players that positively influence bone repair [32, 34, 37, 43], we also modified the chemical composition of SBG to include either ZnO or SrO, followed by their incorporation into PLGA. Previous studies have shown that zinc promotes bone formation in murine osteoblasts and human MSCs [70, 71] and prevents osteoclast activity [72]. Several approaches involving the use of zinc-doped bioactive glasses resulted in increased proliferation and osteogenesis of rat BMSCs and human SaOS-2 cells [39, 73]. Moreover, zinc deficiency can impair extracellular matrix mineralization in osteoblast cultures [74], whereas zinc supplementation has beneficial effects on diabetes-induced bone loss in rats [75]. Notably, osteosarcoma cells are deficient in zinc [76], as zinc has anticancer activity against osteosarcoma cells [77]. Whereas strontium ranelate can protect against osteoporosis [42] and low strontium levels are favorable for osteogenesis [78]. In addition, strontium promotes osteogenesis in MSCs [79], and Sr-doped BG nanoparticles have been shown to facilitate osteogenesis in human BMSCs [44].

We cultured human ASCs on PLGA-based composites containing either unmodified SBGs or modified with SrO or ZnO. ASCs were treated with combined medium supplements with and without fluid flow for 3 days beginning on culture day 1 (Fig. 4a) or cells were first pretreated with rhBMP-2 for 3 days followed by 3-day treatment with combined medium supplements with and without fluid flow (Fig. 4b). As shown in Fig. 4a, the modification of SBGs with ZnO or SrO resulted in significantly elevated mRNA levels of *OPG* and *OC* in human 3-day ASC cultures treated with combined medium supplements and this increase was further enhanced by fluid flow on all modified surfaces except A1-PLGA. Similarly, Fig. 4b shows that in 7-day ASC cultures pretreatment with rhBMP-2 followed by treatment with combined medium supplements was even more effective and was further enhanced by fluid flow. To sum up, the modification of SBGs with ZnO or SrO in the studied PLGA-based composites had an additive effect to the applied culture supplements and media flow resulting in high expression of selected late osteogenic markers. Notably, the composites enriched with ZnO-modified SBGs were more effective in such a culture scheme vs. those containing SrO-modified SBGs. When the latter culture scheme was extended up to 18 culture days (i.e., 3 days of BMP-2 treatment followed by 3 days of combined medium supplement treatment and fluid flow), the human ASC cultures also exhibited the highest expression of *OPG* and *COL1A1* on the ZnO-modified composites (Fig. 5a). Overall, the proposed culture scheme can be repeated in human ASC cultures to maintain high expression levels of osteogenic markers and the PLGA-based composites containing ZnO-modified SBG seem to be the most effective as an osteogenic support.

However, the studies presented in Figs. 1, 2 and 3 indicated some limitations of A1-PLGA composite surfaces as they led to the most distinct responses of ASC cells, despite A1-PLGA surfaces provide an excellent osteoinductive surface for human BMSCs. Upon modification with SrO, this composite surface also provided the poorest results, although ZnO modification led to an overall better response of ASCs on this surface (Fig. 4a, b).

Collectively, our studies indicate that ZnO- and SrO-modified SBGs in PLGA composites may amplify the signaling pathways associated with combined medium supplements (i.e., BMP-2, PD98059, Phenamil) and fluid flow-induced shear stress. The Wnt/β-catenin signaling pathway is one of the key factors during skeletal development and fracture healing, as β-catenin deletion in mice results in osteopenia and prevents terminal differentiation of osteogenic progenitors into osteoblasts [80]. To date, studies have shown that strontium promotes osteogenesis in human umbilical cord MSCs by upregulating β-catenin expression [81] and that zinc activates the Wnt/β-catenin pathway to promote anticancer effects in human osteosarcoma cells [77]. Fluid shear stress has also been shown to induce nuclear localization of β-catenin in murine osteoblasts [82]. To disclose the potential effect of our combined ASC treatment on β-catenin signaling, we have determined that treatment with PD98059 and Phenamil increases phosphorylation of β-catenin at Ser552 (promoting its transcriptional activity) and fluid shear stress has further additive effect on β-catenin phosphorylation; in ASCs cultured on S2-PLGA and SrO-S2-PLGA composites (Fig. 4c). Given that several studies have shown that β-catenin promotes osteocyte formation and bone fracture repair [83] in cells committed to the osteogenic lineage, it is plausible that the transcriptional activity of β-catenin in osteoinduced ASCs (Fig. 4c) may contribute to their faster osteogenic differentiation and acts cooperatively with BMP-2 [84, 85]. Instead, in ASCs cultured on ZnO-S2-PLGA, we observed increased cyclooxygenase-2 (COX-2) expression (Fig. 4c). COX-2 is critically involved in bone tissue repair [86], promotes osteogenesis in MSCs [87] and COX-2 inhibitors such as nonsteroidal anti-inflammatory drugs increase the risk of bone nonunion [88]. We speculate that crosstalk between zinc, BMP and CREB increases transcriptional induction of COX-2 [36, 89, 90], which may be one of the signaling mechanisms responsible for rapid osteoinduction in ASCs cultured on ZnO-SBG-PLGA composites. We also observed increased phosphorylation of CREB in ASC cultures under fluid shear stress on S2-PLGA and SrO-/ZnO-modified composites (the highest level on SrO-S2-PLGA), which may integrate effects of fluid shear stress, COX-2 [91] and BMP-2 [90].

Finally, we also assessed whether human ASC cultures can be prompted to osteogenesis by 7-day preculture on bioactive composite surfaces, followed by their culture on typical plastic tissue culture plates. As presented in Fig. 5b, human ASCs that were prompted to undergo osteogenesis by culture on PLGA-based composites containing ZnO- or SrO-modified SBG produced highly mineralized extracellular matrix, especially upon continuous cell treatment with combined chemical supplements and culture media flow. Notably, in this culture scheme, PLGA-based composites enriched with SrO-S2 provided the highest mineralization levels following 12 days of culture in standard plastic cell culture plates.

Current knowledge regarding the impact of zinc and strontium on the cell response to fluid shear stress is limited as well as the effects of Zn/Sr-doped BGs on ASC osteogenesis. However, some authors have suggested that strontium might positively affect signaling from mechanically stimulated murine osteocytes to osteoblasts [92] and that culture medium perfusion might increase the osteogenic progression of hBMSCs cultured on ZnO-layered scaffolds [93]. Notably, our results show that the expression of bone matrix-related markers can be significantly increased in human ASCs as rapidly as after 3 days of appropriate ASC culture treatment. Moreover, 7 days of ASC preculture on SBG-PLGA composites is sufficient to achieve robust matrix mineralization following 12 days of ASC culture on tissue culture plastic. In other studies, human ASCs cultured on 45S5-based scaffolds exhibited increased ALP activity and collagen synthesis only after approximately 5–6 weeks [11]. Whereas strontium- or zinc-doped BGs increased bone matrix-related gene expression in human osteosarcoma cells after approximately 3 weeks [41, 44].

Potential limitations of this study may involve the heterogeneity of adipose tissue from obese individuals, which results in variability in osteogenic potential [94]. Since our method activates the β-signaling pathway, which is also involved in the transdifferentiation of adipocytes into osteoblasts [95], our method may prove effective even in the stromal vascular fraction (SVF) containing preadipocyte subpopulations. Preclinical studies have shown that systemic administration of ASCs is associated with lower survival and engraftment of ASCs than upon their local administration [94]. Although most clinical trials reported ASCs to be safe and effective, issues regarding engraftment remain debatable [96]. Clinical trials explored local administration of ASCs mainly in wound healing, tendon injury and osteoarthritis [94]. Up to date, only one study in 2015 reported autologous administration of ASCs pretreated in vitro for 3–4 weeks with dexamethasone, sodium ascorbate and sodium dihydrophosphate and supplemented with demineralized bone matrix to form a graft. This has improved bone formation in nonunions with no adverse effects within the next 4 years [97].

For future clinical treatments involving ASCs isolated from adipose tissue, our method can be used either for rapid in vitro differentiation of ASCs into early osteoblasts (3–7 culture days) or into matrix mineralizing osteoblasts (in longer cultures). Furthermore, for local administration osteoblastic cells or mineralizing osteoblasts obtained by our in vitro method can be delivered in suspension, encapsulated in hydrogels or on any suitable implant to support bone regeneration in traumatic bone defects, non/delayed-unions, impaired bone healing or growth disorders etc. Considering fast osteoinduction of ASCs by our method (3–7 culture days) compared to standard methods (3–4 weeks), it seems more feasible in clinical settings for autologous transplantation of osteogenic cells. However, further studies should evaluate the efficacy of this method in vivo with a focus on engraftment and maintenance of bone-forming properties of ASC-derived osteoblasts after in vivo administration.

## Conclusions

In conclusion, we have developed innovative, rapid and effective strategies to differentiate human ASCs into osteoblastic cells. The key elements of such human ASC culture strategies that work well together are the following: PLGA-based bioactive composite surfaces containing ZnO- or SrO-modified SBGs from the CaO-SiO_2_ ± P_2_O_5_ system; osteogenic medium supplemented with rhBMP-2, Phenamil and PD98059; and culture media flow introduced by a standard horizontal laboratory rocker. Of all the studied composites, A1-PLGA (Table 1; Fig. 1e) was the least effective, probably due to its high calcium release [14], which, in turn, could have contributed to the highest NO production that eventually inhibited or slowed down osteogenesis [51]. Treatment of human ASCs with a combination of rhBMP-2, Phenamil and PD98059 effectively enhances their osteogenic progression and works even if the cells are only shortly “conditioned” on the bioactive composite surfaces. The introduction of media flow at the time of ASC treatment with combined medium supplements is the most effective and such treatment can be repeated several times in culture to achieve highly mineralized extracellular matrix. We believe that from all investigated SBG-PLGA composites the best outcomes in ASCs were observed with both S2-PLGA composites modified with SrO or ZnO, when combined with a chemical cocktail stimulation in dynamic culture (Fig. 5). Our results also indicate that the implemented osteogenic strategy contributes to the phosphorylation of β-catenin(S552) and CREB(S133) in ASC cultures on SrO-S2-PLGA, as well as the COX-2 expression in cultures on ZnO-S2-PLGA (Fig. 4d). We believe that these novel strategies for the osteogenic differentiation of human ASCs hold great potential for various bone regeneration therapies. Both the composites and chemically/mechanically treated ASCs, either in combination or separately, may demonstrate suitability for future in vivo applications in the broad bone regeneration-related therapies.

## Methods

### Cells and culture media

ASC52telo cells (ASC; ATCC, SCRC-4000) and normal human ASCs (ATCC, PCS-500-011) were expanded in the dedicated medium (ATCC, Mesenchymal Stem Cell Basal Medium with Mesenchymal Stem Cell Growth Kit and G418). For the experiments, the cells were switched to complete growth medium consisting of 89% MEM Alpha (Thermo Fisher Scientific), 10% FBS Q (Biological Industries) and 1% ZellShield antibiotics (Minerva Biolabs). The cell cultures were maintained in a culture incubator at 37 °C in a 5% CO_2_ humidified atmosphere; the culture media were exchanged every 2–3 days, and the cells were passaged using 0.25% trypsin/EDTA (Thermo Fisher Scientific) before they reached full confluence.

### Experimental cell culture treatments

For the experiments, the SBG-PLGA composites were sterilized by 70% ethanol, rinsed in phosphate-buffered saline, and irradiated on each side with UVC light for 10 min. The composites were then placed in a 24-well culture plate (NEST). To maintain the SBG-PLGA composites at the bottom of the culture wells, sterile 15-ml propylene tubes (NEST) were used to make rings, with each ring measuring 1 cm in height and the diameter of the well in a 24-well culture plate. ASCs were seeded at a density of 10 000/cm^2^ on the SBG-PLGA composites in complete growth medium (as described above). After 24 h, the complete growth medium was exchanged to osteogenic medium, which consisted of complete growth medium supplemented with 100 µg/ml ascorbic acid, 10^− 7^ M dexamethasone and 10 mM β-glycerophosphate (all from Sigma-Aldrich). The standard osteogenic medium was supplemented with recombinant human BMP-2 (100 ng/ml rhBMP-2, ThermoFisher Scientific) or with a combination of 100 ng/ml rhBMP-2 and chemical agents PD98059 (50 µM, R&D Systems) and Phenamil (20 µM, R&D Systems). The medium was exchanged every 3 days. Cells were treated with rhBMP-2 every 3 days starting from day 1. The supplements PD98058 and Phenamil were added on day 4 in 7-day cultures (Figs. 2 and 3) or added in accordance with the specified time frames in Figs. 4 and 5. The culture media flow (1 ml/well) was applied in defined time frames at 3-day intervals (shown in Figs. 3, 4 and 5) with a standard laboratory rocker shaker (MR-1, Biosan) with a tilt angle of 7° and an oscillation frequency of 6 RPM.

### Glass-polymer composites production

Glass-polymer composites were experimentally developed at the Department of Glass and Amorphous Coatings Technology of the AGH University of Science and Technology in Krakow [48]. Composites based on lactic and glycolic acid copolymer (PLGA, Mn = 220 kDa, lactide to glycolide ratio 85:15) were obtained by incorporating bioactive CaO-SiO_2_-P_2_O_5_ or CaO-SiO_2_ glasses into PLGA at 50% by weight. The bioactive glasses were obtained by sol-gel-derived bioactive glasses (SBG) with the compositions listed in Table 1. The average particle size does not exceed 45 μm (sieve analysis).

**Table 1:** The composition of oxides used in bioactive glass synthesis for PLGA-based composite sheets containing 50% Wt. sol-gel bioactive glasses

Bioactive glass	Bioactive glass composition (mol %)				
	SiO_2_	*P*_2_O_5_	CaO	SrO	ZnO
A1	40	-	60	-	-
SrO-A1	40	-	55	5	-
ZnO-A1	40	-	55	-	5
A2	40	6	54	-	-
SrO-A2	40	6	49	5	-
ZnO-A2	40	6	49	-	5
S1	80	-	20	-	-
SrO-S1	80	-	15	5	-
ZnO-S1	80	-	15	-	5
S2	80	4	16	-	-
SrO-S2	80	4	11	5	-
ZnO-S2	80	4	11	-	5

**Fig. 6:**
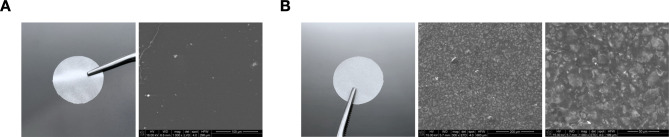
Macroscopic and SEM images of a representative ZnO-S2-PLGA composite at the (**A**) top (cell contact) and (**B**) bottom surface. SEM images show visible bioactive glass particles dispersed in PLGA matrix. SEM scale bars represent (**A**) 100 μm, (**B**) 200 μm (300x magnification) and 50 μm (1000x magnification)

Modified composites with ZnO or SrO oxides were prepared using the sol-gel-derived bioactive glasses (SBG) method from CaO-SiO_2_-P_2_O_5_-SrO/ZnO or CaO-SiO_2_-SrO/ZnO systems, containing 5%mol of SrO/ZnO oxides [57]. The glass compositions can be found in Table 1. The synthesis of glasses utilized the following raw materials: Si(OC_2_H_5_)_4_ (tetraethylorthosilicate, TEOS), OP(OC_2_H_5_)_3_ (triethylphosphate), Ca(NO_3_)_2_•4H_2_O (calcium nitrate), Zn(NO_3_)_2_•6H_2_O (zinc nitrate), and Sr(NO_3_)_2_ (strontium nitrate). The synthesis took place in an environment consisting of ethanol (C_2_H_5_OH, 96%), water, and hydrochloric acid (HCl) acting as a catalyst for hydrolysis and polycondensation processes. The starting solutions were left to gel under ambient conditions and then the resulting gels were dried and heat-treated to 700 °C using an electric oven for an annealing time of 20 h. Similarly to the unmodified composites, the glass powders obtained with a grain size less than 45 μm were introduced into dichloromethane (CH_2_Cl), which serves as the solvent for the copolymer of lactic and glycolic acid (PLGA). Solutions were prepared at a weight ratio of 1:1:53 for bio-glass, polymer, and solvent. Substrates were produced with a weight ratio of 50% bio-glass per dry weight of PLGA copolymer using a solution casting technique. The composite films were dried under cover at 20 °C ambient conditions and subsequently in a vacuum dryer at 800 mbar. All the SBG-PLGA composites obtained have a similar surface microstructure, and the representative macroscopic and SEM images of the top (cell contact) and bottom surfaces are shown in Fig. 6 (more detailed material properties can be found in our previous studies [14, 48, 57]).

### RT-PCR

Total RNA was extracted using TRI Reagent (Zymo Research). Equal amounts of RNA were reverse transcribed using high-capacity cDNA Reverse Transcription kit (Applied Biosystems). PCR amplifications were performed using the StepOnePlus Real-Time PCR Systems (Applied Biosystems). Each reaction mixture contained 50 ng of cDNA, 2–3 µM forward and reverse primers with Sensitive RT HS-PCR Mix SYBR (A&A Biotechnology) or TaqMan probes with TaqMan Universal Master Mix (Thermofisher Scientific). The following probes were used: RUNX2 Hs00231692_m1, SP7 (Osterix) Hs01866874_s1, FOS (Hs04194186_s1), TBP Hs00427620_m1. The sequences of the primer sets (Genomed) that were used are listed in Table 2. Relative expression levels were obtained with the 2^-ΔΔCT^ method.

**Table 2:** The applied PCR primer sequences

	Forward primer sequence (5’-3’)	Reverse primer sequence (5’-3’)
Osteocalcin (*OC*)	AAGAGACCCAGGCGCTACCT	AACTCGTCACAGTCCGGATTG
Bone sialoprotein (*BSP*)	AACGAAAGCGAAGCAGAA	TCTGCCTCTGTGCTGTTGGT
Osteopontin (*OPN*)	TGGAAAGCGAGGAGTTGAATG	CATCCAGCTGACTCGTTTCATAA
Osteoprotegerin (*OPG*)	GTCAAGCAGGAGTGCAATCG	TAGCGCCCTTCCTTGCATT
Osteonectin (*ON*)	GACTACATCGGGCCTTGCAA	GAGTGTGTGCCCACTGAGGAGTCCAAC
Vascular endothelial growth factor (*VEGF*)	GAGTGTGTGCCCACTGAGGAGTCCAAC	CTCCTGCCCGGCTCACCGCCTCGGCTT
Collagen type I (*COL1A1*)	GTCTAGACATGTTCAGCTTTGTGGA	CTTGGTCTCGTCACAGATCACGTCAT
Bone morphogenetic protein 2 (*BMP-2*)	TGCTAGTAACTTTTGGCCATGATG	GCGTTTCCGCTGTTTGTGTT
Noggin (*NOG*)	GCGCTGCGGCTGGAT	AGCACTTGCACTCGGAAATGA
*TBP* (reference gene)	GGAGCTGTGATGTGAAGTTTCCTA	CCAGGAAATAACTCTGGCTCATAAC

### Nitric oxide detection

Nitric oxide was measured by determining of nitrite and nitrate levels in the cell culture medium from 24-hour ASC culture on the SBG-PLGA composites using a commercially available Nitric Oxide Assay Kit (Thermo Fisher Scientific). According to the manufacturer’s instructions, nitrite and nitrate levels were measured at 540 nm using SpectraMax iD3 Molecular Devices reader, and nitric oxide concentrations were calculated.

### Western blot

The whole-cell extracts were obtained using whole cell lysis buffer (Cell Signaling Technology). Protein concentrations were determined by Pierce MicroBCA Protein Assay Kit (ThermoFisher Scientific). Equal amounts (30 µg) of proteins were separated on NuPAGE 4–12% Bis–Tris gels and transferred to PVDF membranes (ThermoFisher Scientific). Membranes were probed overnight with primary anti-human antibodies and then for 1 h with the horseradish peroxidase-conjugated secondary goat anti-rabbit antibodies (Abcam, ab6721). The following rabbit primary antibodies were used at dilutions recommended by Cell Signaling Technology: anti-phospho-ERK1/2 (#9101), anti-phospho-SMAD1/5/8 (#13820), anti-phospho-β-catenin (Ser552) (#5651), anti-COX-2 (#12282), anti-phospho-CREB (#9198). Mouse anti-GAPDH-HRP (#51332) antibodies were used for protein levels normalization. The signal was developed by Western Lightning Chemiluminescence Reagent Plus (GE Healthcare) and the results were generated on CL-XPosure Film (ThermoFisher Scientific).

### Mineralization of the extracellular matrix

Cell cultures were first assayed for viability using The CellTiter 96 AQueous One Solution Cell Proliferation Assay (MTS Assay, Promega). Cell cultures were then fixed with 100% methanol and then stained for 30 min with 1% (water solution) Alizarin Red S (ARS, Sigma). For semiquantitative assessment of the ECM mineralization level, the plates were washed with distilled water, and the ARS was extracted with 5% perchloric acid. The absorbance of the extracted dye was measured at 490 nm using SpectraMax iD3 Molecular Devices reader. The results were normalized to the viable cell number.

### Fluorescence imaging

After 3 days of static or dynamic culture, cells were fixed with 4% paraformaldehyde and Phalloidin-Atto488 (Sigma-Aldrich) was used to detect F-actin cytoskeleton according to manufacturer’s instructions. Cell nuclei were stained with Hoechst (Sigma-Aldrich). Images were acquired with ZEISS Axiovert 5 Microscope.

### SEM imaging

The microstructure of the representative composite was visualized by Nova NanoSEM 200 Scanning Electron Microscope (FEI, Eindhoven, The Netherlands) after coating with carbon. The measurement was carried out in low vacuum conditions with accelerated voltage of 15–18 kV.

### Statistical analysis

All experiments were performed in triplicates, the data were collected as means ± SDs, and the data were analyzed for statistical significance using one-way or two-way analysis of variance (ANOVA) followed by Tukey’s test for multiple comparisons.

## Electronic supplementary material

Below is the link to the electronic supplementary material.

Supplementary Material 1

Supplementary Material 2

## Data Availability

No datasets were generated or analysed during the current study.
