# Supplementary material for: Rapid osteoinduction of human adipose-derived stem cells grown on bioactive surfaces and stimulated by chemically modified media flow
Source: J Biol Eng. 2025 Mar 14;19:23. doi: 10.1186/s13036-025-00491-2 (PMC11908086; doi:10.1186/s13036-025-00491-2)
Supplement: Supplementary file 1 — Supplementary Material 1 [file 13036_2025_491_MOESM1_ESM.docx]

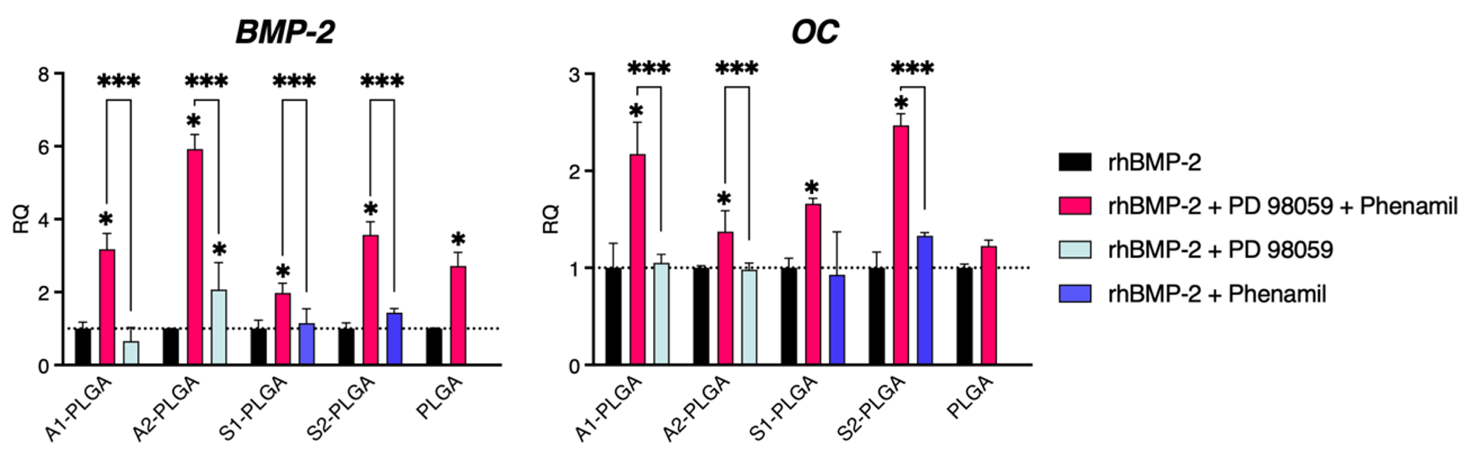


**Supplementary figure 1.** **Cumulative effect of Phenamil and PD 98059 treatment in rhBMP-2 stimulated human ASC cultures.** mRNA levels of osteoblastic markers in 7-day ASC cultures on SBG-PLGA composites. ASCs were cultured in osteogenic medium supplemented with different combinations of 100 ng/ml rhBMP-2, 50 μM PD 98059 and 20 μM Phenamil. Results are presented as relative mRNA expression compared to mRNA levels in cells cultured on the respective SBG-PLGA with rhBMP-2 only (marked as black line at 1). Averages ±SD are indicated. Two-way ANOVA test, *p < 0.05, **p < 0.001, ***p < 0.0001 relative to respective SBG-PLGA with rhBMP-2 or between marked groups. *BMP-2* – bone morphogenetic protein 2, *OC* – osteocalcin.
