# Supplementary material for: Rapid osteoinduction of human adipose-derived stem cells grown on bioactive surfaces and stimulated by chemically modified media flow
Source: J Biol Eng. 2025 Mar 14;19:23. doi: 10.1186/s13036-025-00491-2 (PMC11908086; doi:10.1186/s13036-025-00491-2)
Supplement: Supplementary file 2 — Supplementary Material 2 [file 13036_2025_491_MOESM2_ESM.docx]

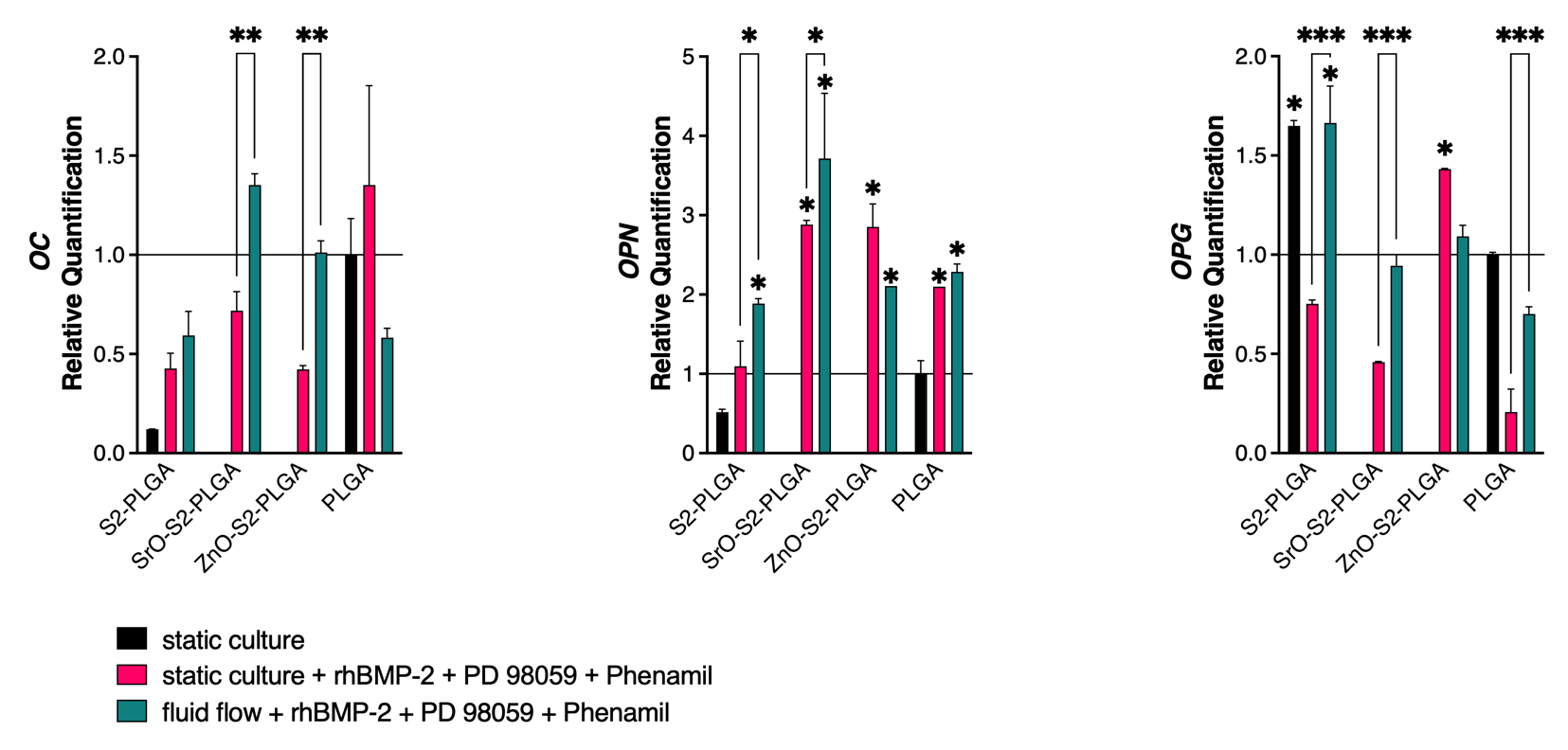


**Supplementary figure 2.** **Verification of the rapid osteoinduction culture method in normal human primary ASCs.** mRNA levels of osteoblastic markers in 3-day osteogenic normal primary ASC cultures on PLGA-based composites containing either unmodified or SrO- or ZnO-modified SBGs. Cells were treated with a combination of 100 ng/ml rhBMP-2, 50 μM PD 98059 and 20 μM Phenamil at the indicated culture times in either static cultures or under fluid shear stress. The treatment setup was as described in Figure 4. Results are presented as relative mRNA expression levels vs. mRNA levels in a control, static culture on PLGA (marked as a black line at 1). Averages ±SD are indicated. Two-way ANOVA test, *p < 0.05, **p < 0.001, ***p < 0.0001 relative to the respective static PLGA control group.
